# Enhancing the Properties of Photo-Generated Metallized Nanocomposite Coatings through Thermal Annealing

**DOI:** 10.3390/nano14020193

**Published:** 2024-01-15

**Authors:** Marine Dabert, Dorina T. Papanastasiou, Loïc Vidal, Samar Hajjar-Garreau, Daniel Bellet, Daniel Lougnot, Lavinia Balan

**Affiliations:** 1 Univ. d’Orléans, Conditions Extrêmes Matériaux Haute Température et Irradiation CNRS UPR 3079, F-45000 Orléans, France; 2 Univ. Grenoble Alpes, CNRS, Grenoble INP, LMGP, F-38000 Grenoble, France; d.t.papanastasiou@gmail.com (D.T.P.); daniel.bellet@grenoble-inp.fr (D.B.); 3 Univ. de Haute Alsace, Institut de Science des Matériaux de Mulhouse, CNRS UMR 7361, F-68100 Mulhouse, France; loic.vidal@uha.fr (L.V.); samar.hajjar@uha.fr (S.H.-G.); 4 Univ. de Haute Alsace, Centre de Recherches sur les Economies, les Sociétés, les Arts et les Techniques CRESAT EA 3436, F-68100 Mulhouse, France

**Keywords:** photo-induced synthesis, metallized silvered coating, reflective conductive film, metal/polymer hybrid, silver nanoparticles

## Abstract

In this work, the effect of thermal annealing on silver nanoparticles@polymer (AgNPs@polymer) nanocomposite coatings was investigated. These photo-generated metallized coatings have a spatial distribution of metal nanoparticles, with a depth-wise decrease in their concentration. During annealing, both structural and morphological variations, as well as a spatial reorganization of AgNPs, were observed, both at the surface and in the core of the AgNPs@polymer coating. Owing to their increased mobility, the polymer chains reorganize spontaneously, and, at the same time, a hopping diffusion process, caused by the minimization of the surface energy, promotes the migration and coalescence of the silver nanoparticles towards the surface. The layer of discrete nanoparticles gradually transforms from a weakly percolative assembly to a denser and more networked structure. Consequently, the surface of the coatings becomes significantly more electrically conductive, hydrophobic, and reflective. The general trend is that the thinner the nanohybrid coating, the more pronounced the effect of thermal annealing on its spatial reorganization and properties. These results open up interesting prospects in the field of metallized coating technology and pave the way for integration into a wide variety of devices, e.g., efficient and inexpensive reflectors for energy-saving applications, electrically conductive microdevices, and printed electronic microcircuits.

## 1. Introduction

Nanohybrid materials are of great interest, due to their versatile and tunable properties. For example, a polymer matrix loaded with metal nanoparticles (MNPs) can offer a wide variety of electrical, optical, or antimicrobial properties by varying the nature of the metal, the morphology of NPs, or the synthesis process [1,2,3]. In this context, many strategies to elaborate metal/polymer nanocomposites have been experimented with, based on either ex situ [4,5,6] or in situ [7,8,9,10,11] synthesis routes. Moreover, in both cases, the spatial organization of MNPs in the nanocomposite structure plays a key role in the properties of nanomaterials, especially for silver-filled materials, which exhibit high electrical conductivity [12].

In this context, Johnsen et al. applied an electrical field to align commercial silver particles that were dispersed in a polymerizable formulation; then, they cured the matrix by heating or irradiating it, which froze their spatial distribution in the material and thus significantly improved the electrical conductivity associated with the silver network [13]. Furthermore, by controlling the electromigration effect, Sandouk et al. generated organized nanowires in the material, hence prompting a decrease in the electrical resistivity of the samples by 5 to 6 orders of magnitude [14].

Thermal post-treatment was also used to increase conductivity in the case of silver nanowire networks; indeed, optimizing junctions goes hand in hand with a decrease in contact resistance between nanowires, as reported by Langley et al. [15]. In general, the thermal annealing process is responsible for an increase in electrical conductivity, due to the decomposition of organic residues and improved coalescence of the MNPs or MNWs (metallic nanowires). This process, which is the main driver of the intimate reorganization of the nanohybrid material, results from a spontaneous tendency to minimize the surface energy of the metal/polymer coating [16,17].

This reorganization increases the compactness of the nanoparticle layer; it induces an increase in the number of conductive contacts between them and, thus, lower junction resistance [18]. Several studies devoted to the coalescence of metal micro-particles [19] or silver nanowire [20] coalescence when subjected to thermal treatment are reported in the literature. Regarding silver–polymer nanocomposites, Fantino et al. [21] printed 3D structures with a liquid photopolymerizable formulation containing silver salts as precursors. In a second step, they induced the photoreduction of metal precursors to metal nanoparticles by annealing the printed samples at 200 °C under vacuum, which resulted in a decrease of one order of magnitude in electric resistivity [21]. Hoeng et al. [22] studied a nanocellulose–silver ink formulation for use in inkjet printers. They first carried out the oxidation of cellulose nanocrystals by TEMPO (2,2,6,6-tetramethylpiperidine 1-oxyl) over a 3 h period before adding the silver precursor and reducing agent. In a similar approach, by heating the final material up to 250 °C, they also reduced the electrical resistivity of the silver nanoparticle-based network by an order of magnitude [22].

Despite the encouraging results from some research groups, the chosen synthesis routes require several steps and, sometimes, long or vacuum-based treatments. In addition, the formulation of inks loaded with large amounts of metal nanoparticles with suitable usage characteristics remains a challenging issue. Additives and solvents are usually required to tailor the viscosity and surface tension and to ensure good colloidal stability, which is not always compatible with the inkjet 3D printing approach.

Other research groups have favored the so-called polyol process, which is typically carried out in a two-step manner [8,18]. In this approach, the reduction of precursor metal salts to silver requires heating to temperatures near the boiling point of the polyol (190–328 °C), a step that can take up to several hours [8].

In a previous work, we introduced an innovative procedure to generate metallized surfaces (Ag or Au) using a single-step photo-induced approach [11,23]. Its originality lies in the nanoassembly of metal particles generated by the in situ photoreduction of precursor ions within a photopolymerizable binder placed under UV exposure for a few minutes. A quite unusual spatial distribution of AgNPs was thus generated, i.e., a concentration profile with a depth-wise gradient shape within the photopolymer. As a result, the surface of the as-synthesized coatings showed a mirror-like appearance similar to that of a silver metallic coating, with varying degrees of electrical conductivity.

In the original version of the process, which was first developed to generate highly reflective surfaces, relatively thick films obtained by drop deposition (typically 300 µm) were used [11]. The disadvantage was that long exposure to actinic light and, above all, large quantities of silver precursor were required, making the process economically unsustainable. In order to make the films produced in this way even more attractive, both in terms of cost and physical properties, i.e., their electrical conductivity, several steps have been taken forward as demonstrated in the present work. In particular, thinner films (10 to 100 µm) requiring much less formulation and lower irradiation doses have been tested, with particular attention to the conversion rate of Ag^+^ ions into the AgNPs participating in the conduction. To achieve the highest conductivity, the effect of post-treatment thermal annealing on the density depth gradient of the nanoparticles of the metallized coatings was carefully evaluated. Its influence not only on the electrical properties but also on other usage properties (reflectivity, wettability, etc.) was analyzed and interpreted from structural studies of the materials via scanning and transmission electron microscopy (SEM and TEM), X-ray diffraction analysis and photoelectron spectroscopy (XRD and XPS), reflection spectroscopy, and contact angle measurements. Throughout this work, coalescence and percolation between nanoparticles appear to be the key phenomena involved in the enhancement of the properties induced by thermal annealing.

## 2. Materials and Methods

*Material synthesis*. Silver nitrate (AgNO_3_) with a purity of >99% and diphenyl (2,4,6-trimethylbenzoyl) phosphine oxide were purchased from Sigma-Aldrich, while polyethylene glycol (600) diacrylate monomer (PEG600DA), with a viscosity of 80 mPa.s at 25 °C, was purchased from Sartomer. All materials were used as received.

*Photo-induced synthesis and thermal annealing.* The photosensitizer, diphenyl (2,4,6-trimethylbenzoyl) phosphine oxide (0.5 wt %), and the metal precursor AgNO_3_ (5 wt %), were mixed with the acrylate monomer (94.5 wt %) under stirring for 60 min at room temperature to obtain a photosensitive formulation [11]. This mixture was applied to a glass slide with a byko-drive applicator at 10 mm/s. The samples were then exposed to UV light for 3 min at 400 mW/cm^2^ in order to achieve the simultaneous photopolymerization and photoreduction of the silver precursor into nanoparticles. Four sample thicknesses obtained with appropriately calibrated bars were investigated (10, 25, 50 and 100 µm). The UV-visible absorption spectrum of the sample that was 10 µm thick shows a characteristic surface plasmon absorption of around 460 nm of as-synthesized silver nanoparticles dispersed in the polymer matrix (see Figure S1).

In a second step, the samples were thermally annealed in air at atmospheric pressure up to 350 °C. To achieve this, the samples were cut into 12 × 12 mm^2^ pieces, and silver-paste contacts were placed on two opposite sides. The samples were annealed using a thermal source associated with a Eurotherm PLC, controlled by LabVIEW. The annealing protocol involved gradually heating the AgNPs@polymer composite samples from room temperature to 350 °C, at a rate of 5 °C/min. Subsequently, the samples were held at 350 °C for one hour, followed by a controlled cooling ramp of 5 °C/min back to room temperature. This treatment was carried out in air at atmospheric pressure. Such a slow and progressive heating regime was chosen to favor the dissipation of the mechanical stress likely to appear in the samples.

The electrical conductivity of various thin Ag@polymer coatings was evaluated through resistance measurements on samples that were used as synthesized and thermally treated, both ex situ and in situ, respectively. For the ex situ measurements, a so-called four-point configuration was used [24]. The in situ measurements were carried out with a frequency of 5 s with a Keithley 2400 sourcemeter, using a two-point configuration and a very low voltage (0.2 V), to avoid any risk of electrical degradation.

The impact of the thermal treatment on the wettability of the coatings, measured via the contact angle of water drops deposited on the surface, was determined using a DSA100-KRUSS goniometer.

*Material characterization.* Photo-induced reactions were performed with a Heraeus Noblelight America LLC UV lamp with an H bulb, with the actinic beam delivering a fluence of 400 mW/cm². The reflectance spectra were obtained using a Thermo Fisher Scientific Evolution 200 UV-Vis spectrophotometer equipped with an integrating sphere. The morphology and spatial organization of the nanoparticles inside the polymer matrix were characterized by transmission electron microscopy (TEM). Measurements were performed at 200 kV using a Philips CM200 instrument with a LaB6 cathode.

Scanning electron microscopy (SEM) investigations were performed with an XL30 Philips scanning electron microscope (at 100,000× magnification). X-ray diffraction experiments were performed with a Philips X’Pert MPD diffractometer equipped with a Cu Kα radiation of λ = 0.1546 nm, running at 40 kV and 40 mA. A VG Scienta SES 200-2 spectrometer was used for the X-ray photoelectron spectroscopy (XPS) experiments, which were performed at *p* < 10^−9^ mbar via a monochromatic Al-K_α_ X-ray source (energy: 1468.6 eV). The depth measures were approximately 8 nm at normal incidence. The wide-scan and high-resolution spectra were collected with pass energy settings of 100 eV and 500 eV, respectively. The deconvolution of the spectra was carried out with a Gaussian (70%)–Lorentzian (30%)-shaped peak, or an asymmetry function in the case of Ag(0), using the 2.3.18 version of CasaXPS software after subtracting a Shirley-type background. The atomic percentages were determined by taking into account the transmission factor of the spectrometer, the mean free path, and the sensibility factor of each type of atom investigated [11].

## 3. Results and Discussion

### 3.1. Evolution of Electrical Conductivity under Thermal Annealing

**Figure 1:**
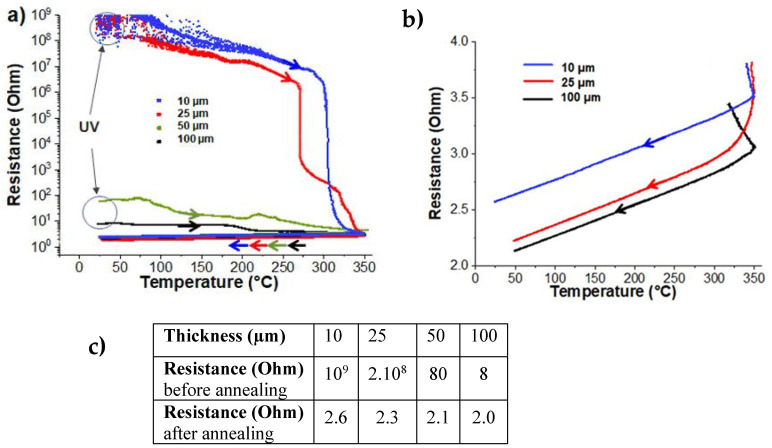
(**a**) Electrical resistance changes during the thermal ramping up to 350 °C and back to room temperature for samples with increasing thicknesses of 10, 25, 50, and 100 µm. (**b**) Zoom in of the section of (**a**) showing the evolution of the bulk resistance of three representative samples during the cooling step and (**c**) the measured bulk resistances of the samples before and after annealing.

During the annealing process, low heating/cooling rates were chosen in an attempt to avoid those stresses potentially induced by higher heating/cooling rates (see Section 2). Initially, the evolution of electrical conductivity during annealing was investigated, as it is a crucial property influencing the significance and potential applications of this type of nanocomposite coating in various fields. The electrical characteristics of Ag@polymer coatings obtained after UV synthesis, also called “UV”, were observed to be strongly dependent on the sample thickness. As shown in Figure 1a,b, over a series with a thickness ranging from 10 to 100 µm, the general trend was that the thicker the initial film, the lower its electrical resistance. The coatings that were 10 and 25 μm thick were definitely non-conductive (with resistance in the range of 10^8^–10^9^ ohms), while the coatings that were 50 and 100 μm thick were conductive, with resistances of 80 and 8 ohms, respectively.

Electrical conductivity is clearly related to the characteristics of the silver NPs constituting the upper layer of the nanomaterial coating, specifically, their morphology and the compactness of their spatial reorganization. A key parameter is the AgNP size compared to the average electron-free path (Λ) in silver bulk, which is close to 50 nm at room temperature [25]. Particle nanoassemblies with sizes smaller than Λ are known to exhibit high electrical resistivity, due to enhanced electron surface scattering, and such behavior is particularly reported in the literature for AgNW [16]. Thus, the small-sized particles constituting these upper layers, when combined with the low percolative nature of their stacking, may account for the very high electrical resistance of the thinnest coatings (10 and 25 µm).

Figure 1a shows the real-time evolution of the bulk resistance of the four samples that were subjected to thermal annealing. Two very different behaviors appear immediately. The resistance of two of them, the thick coatings that were initially identified as good conductors, decrease more or less steadily from room temperature to 350 °C. No real anomaly or singularity along this decrease seems to evoke any particular event occurring in a specific temperature range. In contrast, the curves showing the evolution of the resistance of the other two samples, where the thinnest coatings were initially non-conductive, seem more informative. They consist of three parts: a steady decrease of limited amplitude between room temperature and about 300 °C, followed by a drastic decrease over a narrow range of a few degrees and, finally, a phase of progressive stabilization up to 350 °C.

Two types of transformation that are induced by thermal activation are involved in the restructuring of these hybrid nanocomposites. In the first, the temperature increase must induce a redistribution of the organic binder adsorbed on the surface of particles, resulting in an improvement in the percolation quality of the silver network through a spatial reorganization of the small amplitude of the junction zones. At higher temperatures, a hopping diffusion of the particles caused by the minimization of surface energy promotes coalescence and the creation of a denser network of junctions, which leads to the drastic enhancement of electrical conductivity [26,27]. The network of discrete nanoparticles is converted from a percolative structure to a more continuous one, the electrical characteristics of which approach those of thin bulk silver films.

For metals over a wide temperature range, the relationship between electrical resistivity and temperature is nearly linear (while admitting that the dimensions do not change significantly when the temperatures increase up to 350 °C). Indeed, as can be clearly seen in a zoom-in of the corresponding part of Figure 1a, during the cooling step, the bulk electrical resistance decreases linearly with temperature (Figure 1b). When the resistance of a metal sample is measured at temperature T_0_ and, then, T_0_ + ΔT, the value for β can be derived as follows:

$$\beta =\frac{∆R}{R_{0}\cdot ∆T}$$

where β is the temperature coefficient of electrical resistance, and ΔR is the resistance change (relative to R_0_) that is associated with a temperature change of ΔT.

From the results of Figure 1b, the temperature coefficient of bulk resistance is calculated and ranges from 1.0 × 10^−3^ K^−1^ (10 µm) to 1.3 × 10^−3^ K^−1^ (100 µm). These values are substantially lower than those reported in the literature (i.e., for bulk Ag thin films [28] (3.8 × 10^−3^ K^−1^), for AgNWs [29] (3.32 × 10^–3^ K^−1^), for AgNPs films [30] (1.56 × 10^−3^ K^−1^), or even for AgNPs networks [31] (2.2 × 10^−3^ K^−1^)).

The differences between solid silver and the networks of assembled AgNPs obviously derive from their inner structure. For the AgNPs, this means a random stacking of crystalline aggregates that are connected by tenuous junctions of various cross-sections, with an extremely high number of defects and grain boundaries. Moreover, in the specific case of nanohybrid systems, the presence of voids (open spaces) and a significant quantity of organic residues stemming from the polymer binder may explain why the temperature coefficient of resistance is much lower than that of bulk silver [32].

**Figure 2:**
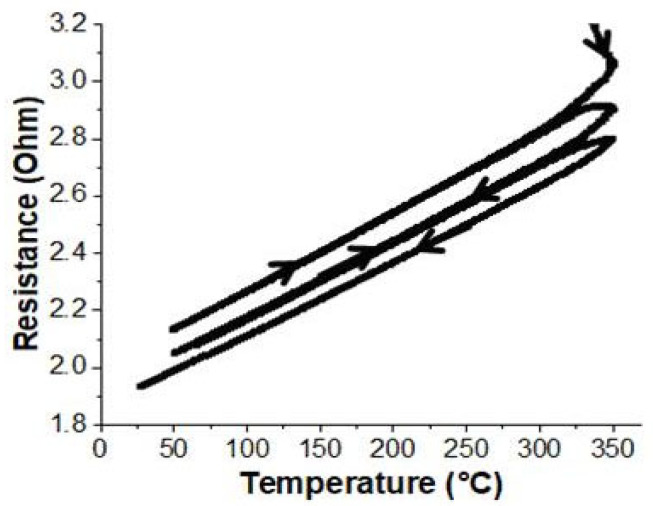
Real-time evolution of electrical resistance during three repeated similar thermal ramp cycles for a sample that is 100 µm thick. Arrows indicate the direction of temperature evolution with time.

In order to ensure that the reference protocol was sufficient to achieve long-term stability after one annealing cycle, a 100-µm-thick sample was consecutively subjected to three thermal cycles. The in situ monitoring of the sample’s bulk resistance throughout this treatment is reported in Figure 2. As is demonstrated, from one cycle to the next, the temperature coefficient of resistance remained constant and within the experimental error. Above 320 °C, additional annealing cycles induced a very slight decrease in bulk resistance. This is most likely related to the pyrolysis of organic residues, which improves the quality of the junctions between the metal aggregates and, consequently, their degree of percolation.

### 3.2. Transmission Electron Microscopy

**Figure 3:**
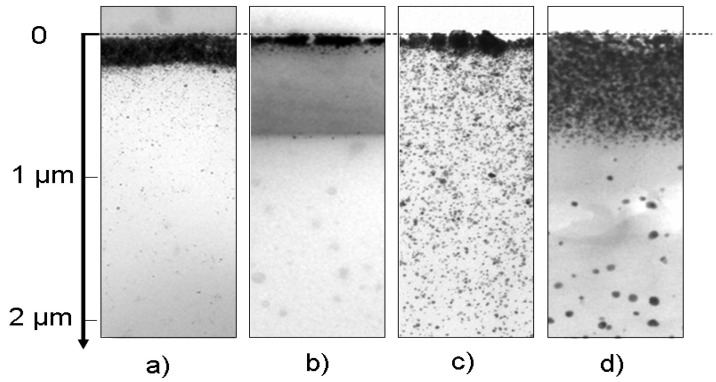
Cross-sectional TEM micrographs showing the morphology of two representative samples; 10 µm sample before (**a**) and after (**b**) annealing; 100 µm sample before (**c**) and after (**d**) annealing.

From this first study, it is clearly evident that thermal annealing definitively enhances the electrical conductivity of the coatings obtained by photochemical treatment. However, behind the dramatic improvement of this macroscopic property, a deep reorganization of the inner structure of the nanocomposite material is very likely to be hidden. With a view to identifying the nature of the phenomena responsible for better electron circulation and thus going further in terms of comprehension of the effects of thermal annealing, a TEM study has been performed on the cross-sections of a microtome-cut Ag@polymer coating. For comparison, Figure 3 shows two pairs of TEM images corresponding to two samples (10 and 100 µm thick) before and after annealing.

Qualitative observation of the as-synthesized samples showed a dense layer of elementary AgNPs and aggregates embedded in polymer binder, which formed in the upper part of the coating generated by UV synthesis. Interestingly, a general trend emerged, i.e., the thickness of this top layer of metal, which accumulated near the surface of the samples, increased in tandem with the AgNPspolymer nanocomposite coating’s thickness, both for those shown in the figure and for others—not shown—corresponding to intermediate thicknesses. The underlying layer, which constituted the core of the coating, appeared much less dense. It exhibited a typical distribution of AgNPs with a negative gradient profile, i.e., a concentration gradually decreasing from the continuous silver surface layer down to the depths of the coating. The deepest part of the coating seemed to be completely free of any Ag particle.

Upon thermal annealing, a small weight loss (0.37%) was observed for all samples, probably due to a slight degradation of the polymer, which is associated with the release of volatile oxidation products. Regardless of the thickness of the coatings, the TEM images consistently show profound depth-wise reorganization. The first striking observation is the densification and the thickening of the surface layer, where more compact and interconnected aggregates and strings of AgNPs have formed. In agreement with Southward’s observations on Ag/polyimide films, the atomic density of Ag increased as the interface was approached [9].

At the same time, the underlying layer in the samples appeared to be more or less depleted of nanoparticles. The few of them that remained had aggregated and concentrated near the underside of the continuous layer at the surface of the samples. It thus appears reasonable to postulate that some of the particles that were initially presented have migrated towards the upper surface to clump with the bulk metal layer and increase both its density and thickness. These two domains appear clearly delineated. It should also be noted that in the depths of the thickest samples, some rare, isolated particles were also present.

Such a spatial redistribution of the nanoparticles in the depths of the sample was also reported by Southward et al. [33] in polyimide films, but without further comment. Thermal treatment induces both an increase in the mobility of polymer chains and a weakening of the stabilizing effect of metal nanoparticles by the polar groups carried by them. The conjunction of these two effects favors the migration of AgNPs, which spontaneously redistribute throughout the thickness of the coatings. Moreover, this phenomenon is dependent on the size of the nanoparticles; the smaller they are, the easier the migration is.

Table 1 summarizes all the observations concerning the effect of annealing on the thickness of the compact layer at the surface of a series of samples of increasing thickness. As an indication, the thickness of the silver metal layer that would be formed by the exhaustive reduction of all the Ag^+^ ions in each of these samples is also shown. For a thickness of 10 µm, it can be seen that after annealing, almost all the ions have been reduced, and the corresponding NPs have migrated to the surface layer. The small thickness of the sample has allowed the diffusion of all the NPs from the sub-layer to the surface.

However, in a sample that is 100 µm thick, as shown in Figure 3, the observations are very different. Since the diffusion times vary as a square function of distances, a fraction of the NPs in the depths of the sample were too far from the surface to have a chance to reach it. These NPs then experienced another fate; they diffused towards some particles entangled around the meshes of the 3D polymer network because of their particularly large size. The latter then captured the neighboring NPs and grew via coalescence to finally form a few, very large, isolated particles that are visible in Figure 3 (75 < d < 150 nm diameter), in a process that is driven by the reduction in local volume free energy.

**Figure 4:**
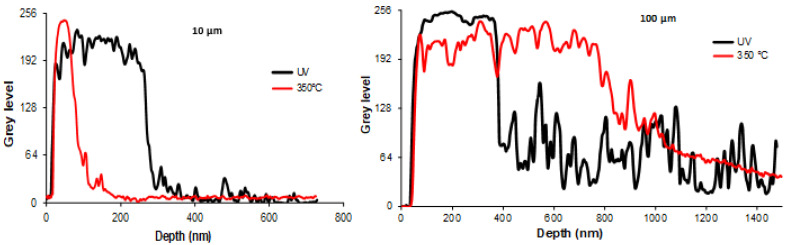
Transmission electron microscopy (TEM) images of the cross-section of the Ag@polymer coatings: the gray level is plotted versus the depth, normally, to the surface; 10 µm (**left**) and 100 μm (**right**) samples; the lines show UV synthesis (black) and 350 °C annealing (red).

Thus, from a combination of all these findings, it appears that the thickness of the compact silver layer forming at the surface of the samples first increases with the thickness of the coatings. Schematically, the higher the amount of available Ag^+^ ions, the more they tend to accumulate at the surface to form this compact layer (Figure 4). However, beyond 25 µm, the phenomenon reverses because the limiting factor becomes transport via diffusion. As the thickness of the coating increases, the nanoparticle front does not have sufficient time to reach the surface layer before the polymer binder vitrifies (Figure 4).

The curve showing the evolution of the thickness of this layer after thermal annealing continuously increases with the thickness of the coatings. Indeed, heating the samples over long periods releases a fraction of the nanoassemblies that are trapped or entangled in the polymer binder and allows those that are not too far from it to finally reach the surface layer. Two interesting additional points deserve comment: first, as the thickness of the coatings increases, the compactness of the surface layer decreases; this is probably due to the difficulty of driving the polymer binder out of this region because of its highly cross-linked structure. The second additional point is the presence in the depths of those samples that were 100 µm thick, representing a small population of large particles that have coalesced in the same way as the compact surface layer (Figure 3 and Figure 4).

### 3.3. Scanning Electron Microscopy

**Figure 5:**
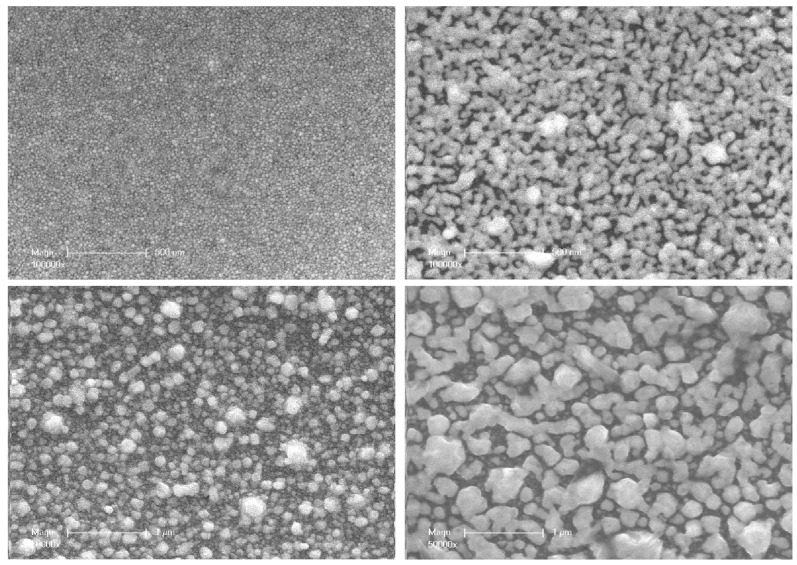
SEM images of the coating surface ((**above**): 10 µm; (**below**): 100 µm; (**left**): before annealing; (**right**): after annealing)).

In order to characterize the surface layer of the nanocomposite coating in more detail, additional SEM studies were performed on both the as-prepared and the annealed samples (Figure 5).

Visual observation of these pairs of SEM images shows that the size of particles on the outer surface of the compact layer increases significantly after annealing, with a broad size dispersion. Moreover, as their sizes increase, their shapes become less and less regular. After UV synthesis, the average size of the nanoparticles at the outermost surface of the set of samples used for TEM investigations ranged from 18 to 68 nm (Table 2). The increase ratio in average particle size with thermal annealing goes from a factor of four (18 to 68 nm) in the thinnest coating (10 µm) to three (68 to 204 nm) in the thickest coating (100 µm).

**Figure 6:**
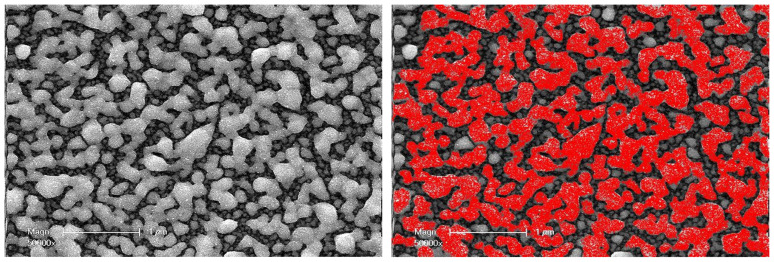
SEM image of the surface of a 50 µm sample after annealing, and the same image with the incompletely coalesced aggregates highlighted in red.

This increase in particle size during thermal annealing is most likely related to the coalescence phenomenon. This corresponds to the fusion of two nanoparticles; it is associated with a reduction in the ratio of the outer surface of these two particles to their total volume. The coalescence thus contributes to a reduction in the internal energy of the system (interfacial free energy). However, some of the SEM images clearly show that groups of nanoparticles have come together, not only to form larger and more or less spherical particles but also to form much more extended shapes. They have formed strings that are simply stuck together and are reminiscent of a sintering process (Figure 6). In reality, it is rather a spatial reorganization of a metastable set of nanoparticles subjected to opposite constraints: the stabilizing effect of the polar functions carried by the polymer network, which keeps the particles apart, and the instability resulting from a high surface-to-volume ratio that tends to minimize the effect. However, the imbalance between these constraints is not sufficient to cause coalescence.

Southward et al. observed such behavior when heating a non-conductive surface (Ag/polyimide films), from 300 °C to 340 °C, to form a conductive surface [7]. Moreover, the spherical Ag nanoparticles aggregated into more elongated shapes, as also shown by Bhattacharyya et al. [34] and, more recently, by Zouari et al. [35], which eventually created the Ag network found near the upper surface of the nanocomposite coating.

At this point, the information gained from the SEM and TEM investigations elucidated the evolution of the electrical properties of Ag@polymer coatings under the effect of thermal treatment at 350 °C. The annealing process resulted in the formation of a compact conductive metal layer, even in the case of coatings that were 10µm thick, via the combination of two phenomena: (i) surface coalescence between NPs, which creates extra conduction paths (see Figure 5); (ii) the migration and coalescence of NPs (see Figure 3 and Figure 4). This results in a general enhancement of the percolating character of the network that was formed by the AgNPs, a character that is even more noticeable near the upper surface of the coating.

**Figure 7:**
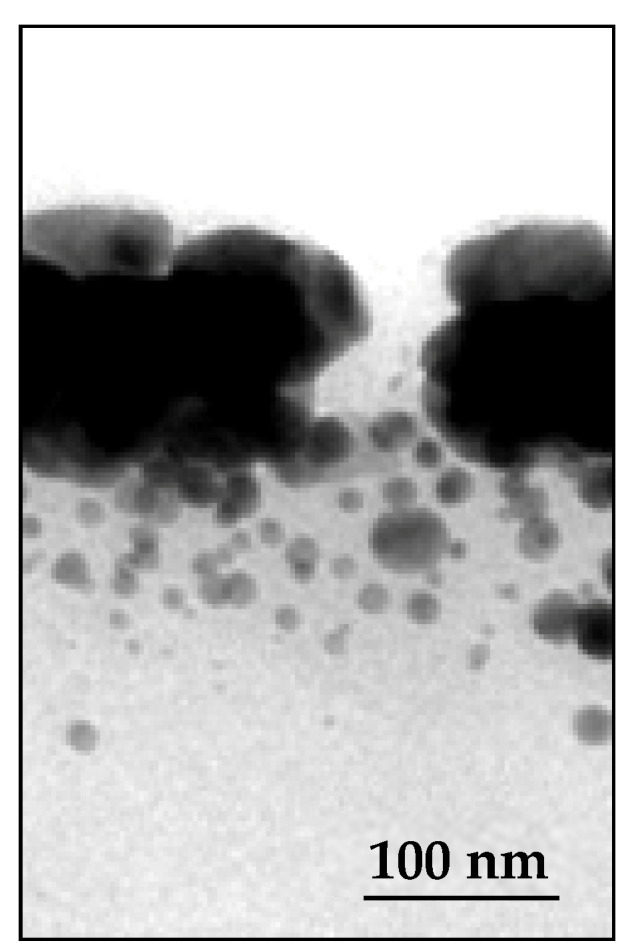
TEM micrograph of the cross-section of a crack in the silver layer on the surface of a 10 µm sample that had been annealed.

Electron microscopy investigations have raised another point about the relationship between the inner structure of AgNPs@polymer composites and their electrical conductivity. It concerns the consequences of the volume shrinkage induced during annealing. When a compact Ag layer is formed by the coalescence of a large collection of NPs at the surface of a nanocomposite coating, relaxation of the mechanical stress associated with shrinkage can only occur via dimensional reduction. In the thickness direction, contraction is impossible because of the incompressibility of the surface layer, which is very rich in MNPs. Conversely, its area can be considered as infinite compared to its thickness; because of the strong interactions that occur with the underlying polymer layer, it cannot constrict either. There is, therefore, no way to dissipate this volume constraint. The only possible resolution is, then, the local lack of cohesion of the surface layer and the creation of a crack, as shown in Figure 7. However, this problem, which can adversely affect the bulk electrical conductivity, was only observed for the thinnest coatings (10 and 25 µm).

### 3.4. X-ray Diffraction Analysis

**Figure 8:**
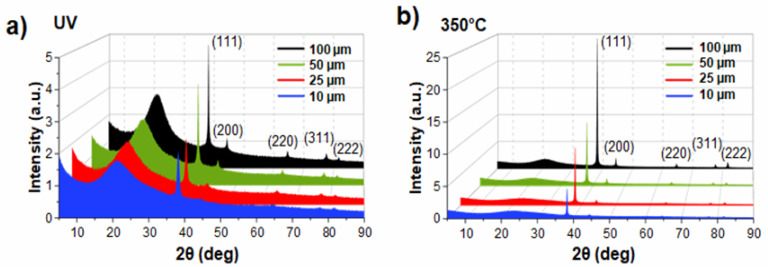
X-ray diffraction (XRD) analysis: (**a**) X-ray diffraction patterns of the Ag@polymer samples after synthesis (UV); (**b**) the samples after annealing (350 °C).

To further characterize the photogenerated coatings, both before and after thermal annealing, X-ray diffraction (XRD) was used to analyze their crystallinity and to reveal those changes induced by thermal post-treatment (Figure 8). As shown in Figure 8a, the XRD diffraction patterns of the as-synthesized samples exhibit the following characteristic crystallographic planes of face-centered cubic (FCC) silver: (111), (200), (220), (311), and (222). The broad peak of 2θ near 25° is related to the amorphous acrylate polymer [23]. After thermal annealing at 350 °C (Figure 8b), the general appearance of the diffraction patterns remains the same, with the same broad peaks due to the amorphous polymer binder, and the same crystallographic planes of Ag. However, the latter show proportionally much more intense peaks. This clearly indicates a very significant improvement in the degree of crystallinity of AgNPs with this treatment.

Four main X-ray peaks are considered when evaluating the degree of crystallographic orientation: (111), (200), (220), and (311). The (222) peak, which is a harmonic of (111), can be ignored. A texture analysis was performed quantitatively via the Harris method, which is commonly used for polycrystalline thin films [36,37] (see the Supplementary Materials for more information on this analysis). The main finding is the existence of a preferential orientation of the crystallites along the (111) planes that is more pronounced after thermal annealing (Figure S2), regardless of the thickness of the samples. These findings can be interpreted as follows: FCC metals, such as Ag, exhibit a preferential (111) orientation since the (111) plane is associated with the lowest surface energy and the highest atom packing density [38]. This trend holds for polycrystalline Ag thin films, in which the crystallographic texture depends on multiple parameters: deposition conditions, the nature of the substrate, film thickness, or post-deposition annealing [39,40,41]. The latter generally leads to an increase in texture on the (111) plane for thin silver films [41]. Indeed, thermal post-annealing and the ensuing spatial reorganizations minimize the total energy of these systems, i.e., the balance between the surface energy and the elastic deformation energy components. The increase in the texture coefficient following annealing in the AgNP arrays that are studied in this work confirms that they behave similarly to polycrystalline thin films; they reorganize in the immediate vicinity of their outer surface, and the 111-face-terminated nanoparticles reorient to align with the surface plane.

### 3.5. X-ray Photoelectron Spectrometry Analysis

**Figure 9:**
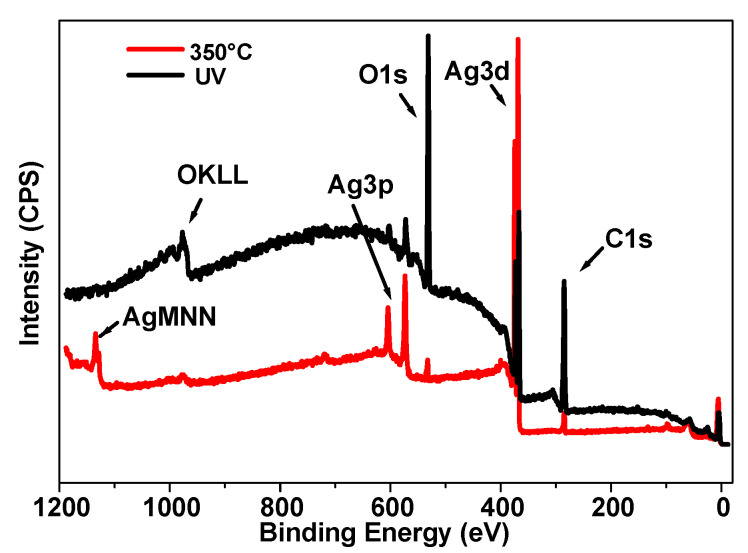
XPS spectra of the external surface of a 10 µm sample, for both the as-synthesized sample (in black) and after annealing (in red).

The thinnest sample (10 µm) of the series under study was analyzed by XPS to investigate its surface chemistry before and after annealing. As shown in Figure 9, the general appearance of the spectrum after UV synthesis demonstrates an important increase in the background, which comes from the inelastically scattered electrons. The electrons that are excited by X-rays travel a certain distance before escaping the surface. During this transport, some electrons lose energy by inelastic scattering and end up at a lower energy level in the spectrum. This energy loss is more important when the electrons come from deeper layers [42,43,44]. This is indicative of a migration of AgNPs to the surface after the 350 °C treatment, which is consistent with the TEM images (Figure 3) that also reveal an important change in structure in the depths of the nanomaterial.

The major elements that were found were carbon, oxygen, and silver. The carbon and oxygen originated mainly from the polymer matrix. Concerning silver, two states were present: its zero state and a very weak peak attributed to Ag oxide, as can be seen in Figure S3. Moreover, the latter state was only observed before thermal treatment, probably due to a slight oxidation of the surface of the photo-generated coating when kept in the air. It is interesting to note that the Ag/C ratio rose from 0.1 to 2.1 upon thermal annealing; this confirms the redistribution of these elements to the first nanometers of the surface of the sample. Furthermore, the possible decomposition of the polymer binder [35] could also indirectly contribute to the increase in the relative ratio of Ag in the surface layer.

Consequently, both the XRD and XPS characterizations show the structural reorganization of the AgNPs at the surface and inside the polymer due to thermal annealing. The XRD measurements clearly indicate that heating the samples to 350 °C increases their crystallinity and texturing along the (111) planes, while the XPS analysis corroborates the evidence of the TEM images (Figure 3), i.e., a decrease in the AgNP gradient and a denser top metal layer; moreover, this shows that the zero state of Ag is preserved after thermal treatment.

### 3.6. Reflectivity of the Samples

**Figure 10:**
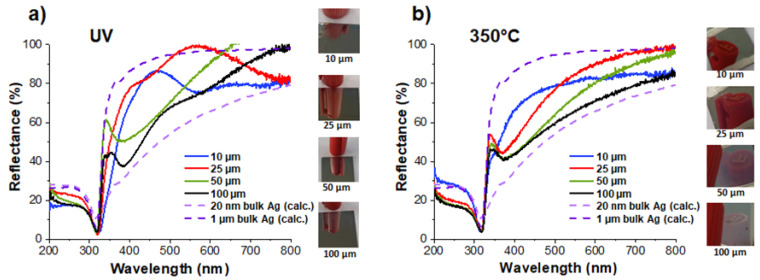
Total reflectance spectra of metallized coatings of various thicknesses: (**a**) as-synthesized samples seen by UV and (**b**) after annealing at 350 °C. The calculated reflectance spectra are shown for 20 nm and 1 μm thick bulk silver layers on an acrylic substrate (dotted lines).

On the whole, upon visual observation, the as-synthesized samples exhibited reflective top surfaces with a good mirror effect (Figure 10). The reflectance spectra have been ascertained over a range of 200 to 800 nm. For comparison purposes, two additional curves have also been inserted in Figure 10, which correspond to the reflection spectra of two bulk silver layers (20 nm and 1 µm) on an acrylic polymer substrate. These spectra were calculated using online freeware [45]. As can be seen in Figure 10a, the reflectance spectra of four samples with a total thickness varying from 10 to 100 µm can be inserted between these two extremes. Their general shape in the mid-UV range is as expected, but not so much beyond 350 nm and throughout the visible. The question arises as to what corresponds to the shape of the reflectance spectra in this range. Two hypotheses can be made: the first one is the possible contribution of the absorption associated with plasmon resonance. The reflecting surface is made of spherical AgNPs with sizes ranging from about ten to a hundred nanometers (Table 2); their plasmon absorption is, therefore, localized in the visible range, with a maximum that is more redshifted and an absorptivity that is weaker as their size increases. However, as the size distribution of the particles on the surface is not known with precision, it is difficult to push further forward in the exploration of this hypothesis. Moreover, nothing excludes the possibility that interference phenomena, caused by a coupling of the reflections on the air/nanocomposite and nanocomposite/polymer diopters, may add to the plasmon absorption.

After annealing, the same four samples showed little change in their visual appearance. The reflectivity of the thinner samples (10 and 25 µm) did not seem to be affected; the mirror effect and the quality of the gloss remained the same. On the other hand, the surface appearance of the thickest samples (50, and especially 100 µm) became milkier, which suggests the appearance of a significant diffuse reflection component. This is not surprising when we know that after annealing, the size of the particles on the surface became several hundred nanometers and at the same order of magnitude as the visible wavelengths.

The corresponding reflectance spectra after annealing are plotted in Figure 10b. Unlike the as-synthesized samples, their general shape appears more conventional. The influence of absorption due to plasmon resonance is clearly visible as a depression in the near-UV. The minima of this depression, which correspond to the maxima of the plasmon resonance, are located at approximately 355 nm for the 10 µm sample (more of a shoulder than a minimum), 365 nm for the 25 µm sample, and 380 nm for the 50 and 100 µm samples. Furthermore, a comparison of these reflection spectra with the (calculated) reflection of a 100 µm solid silver layer suggests that absorption due to plasmonic resonance would extend quite far into the visible spectrum. This would be consistent with a population of silver particles that are very heterogeneous in size and that experience an inhomogeneous environment, with air on one side and an annealed polymer binder on the other.

In any case, it is very remarkable that from samples with a total thickness of between 10 and 100 µm, it is possible to create and spatially arrange metallic nanoparticles by the action of light and thermal annealing to build continuous nanometric layers that are endowed with both high electrical conductivity and high reflectivity. Thus, as can be seen in Figure 10b, a coating of only 10 µm has a constant reflectivity of about 80% at between 500 and 800 nm. One of the points worth highlighting is the very limited amount of Ag involved in this type of sample, consisting of about 50 mg/cm^2^ of conductive and reflective coating. Given the high cost of bulk Ag, there should be genuine interest in this process.

### 3.7. Wettability

**Figure 11:**
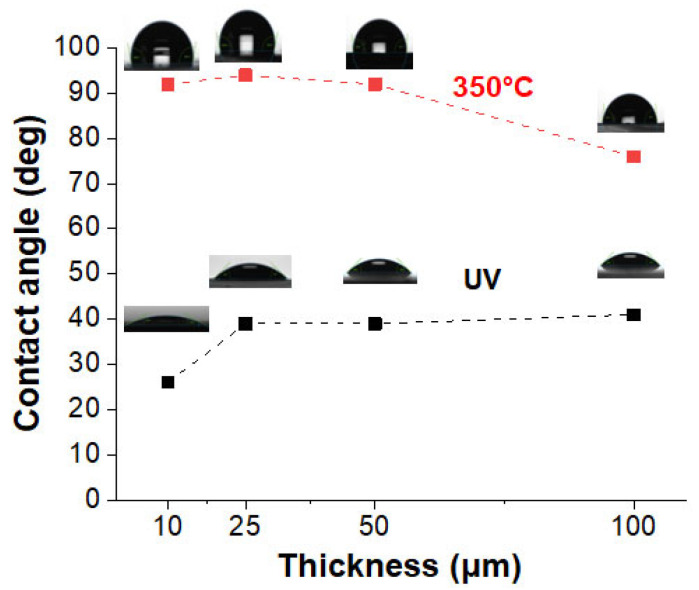
Contact angles and photographic images of the deposited water droplets on metallized coatings of various thicknesses: UV synthesis (black dots) and after thermal annealing at 350 °C (red dots).

Among the various usage properties of this type of coating, surface properties are likely to bring some added value. Wettability was evaluated before and after the thermal step, in order to assess the influence of the reorganization that is induced by this treatment. For this purpose, the contact angles of water droplets on the metalized coatings were recorded and are presented in Figure 11, along with the photos illustrating the droplets on the sample surfaces. Thermal annealing appears to be an extremely effective treatment to reduce the wettability of these metallized coatings. Whatever the thickness being considered, the contact angles increased dramatically compared to those for the as-synthesized samples. Thus, for the 10 and 100 µm samples, the contact angle increased from 25° to 92°, and from 40° to 79°, respectively.

These changes in wettability can be explained by the coalescence phenomenon that was induced by the thermal treatment. The annealing treatment reduced the surface energy and allowed the creation of a more compact AgNP network, with an increase in surface roughness (as shown by the TEM and SEM investigations (see Figure 3 and Figure 5)) and more pronounced hydrophobicity. It is a well-known fact that materials with low surface energy and high surface roughness demonstrate a higher contact angle (wettability reduction) [46]. At the same time, the presence of interparticle voids on the surface leads to heterogeneous wetting, showing a variable contact angle with water depending on the relative importance of these voids and the apparent roughness. This could explain why, on the whole, annealing causes a clear increase in contact angles, but their dependence on sample thickness does not seem to be solely influenced by the surface particle size. In any case, it is important to state that after annealing, the surfaces of the thinnest samples remained at the limit of a hydrophilic character (intermediate–wet), while the thickest (100 µm) remained clearly hydrophilic.

## 4. Conclusions

The photoinduced approach used herein allows the fabrication of metallized Ag@polymer coatings in a single step, with an unusual gradient-shaped distribution of nanoparticles across the thickness of the samples. The present work was aimed at evaluating the potential of thermal annealing to boost the electrical conductivity of such samples. Thermal annealing, when applied after synthesis, induces important structural and morphological changes in the samples. More precisely, this treatment induces a depth-wise redistribution of AgNPs from the core of the polymer matrix towards its surface, together with their coalescence and networking.

The AgNP network thus created near the surface significantly improves the electrical conductivity of the samples. This highlights the close correlation between the spatial organization and inner structure of the material (e.g., the enhancement of the (111) crystallographic texturing) and its properties. At the same time, the reflectivity characteristics of the studied materials are only slightly affected by heat treatment. Finally, the thermally induced reorganization of the AgNPs leads to a drastic increase in the hydrophobic character of the samples’ surface. The thinnest coating (10 µm) offers the best compromise between all the above-studied properties: high reflectance (75%), low electrical resistance (2.6 ohms), and a significantly hydrophobic character (a contact angle with water of 92°). For these reasons, these multifunctional coatings are promising candidates for use as electrically conductive coatings, in low-cost reflectors, or for applications in the field of printed circuit boards.

## Figures and Tables

**Table 1:** Thickness of the dense silver layer at the surface of the coatings before and after annealing.

Thickness (µm)	Before Annealing (nm)	After Annealing (nm)	Compactness of the Top Silver Layer after Annealing	Theoretical Thickness (nm)
10	262 ± 20	58 ± 23	Very high	48
25	700 ± 30	205 ± 18	High	120
50	488 ± 35	640 ± 25	High	240
100	343 ± 50	785 ± 40	Medium	480

**Table 2:** Average sizes of the silver particles, as derived from SEM images before and after thermal annealing.

Thickness of the sample (µm)	10	25	50	100
Size of AgNPs before annealing (nm)	18 ± 6	38 ± 14	55 ± 23	68 ± 29
Size of AgNPs after annealing (nm)	68 ± 33	133 ± 58	176 ± 80	204 ±129

## Data Availability

Data are contained within the article and Supplementary Materials.

## Supplementary Materials

The following supporting information can be downloaded at: https://www.mdpi.com/article/10.3390/nano14020193/s1, Figure S1: UV-vis spectrum of a 10 µm Ag@polymer coatings after UV synthesis; Figure S2: additional XRD information and Figure S3: XPS analysis with detailed peaks of Ag, O and C elements.
