# Supplementary material for: Enhancing the Properties of Photo-Generated Metallized Nanocomposite Coatings through Thermal Annealing
Source: Nanomaterials (Basel). 2024 Jan 15;14(2):193. doi: 10.3390/nano14020193 (PMC10818463; doi:10.3390/nano14020193)
Supplement: Supplementary file 1 [file nanomaterials-14-00193-s001.zip › nanomaterials-2823135-supplementary.pdf]

Supporting information

Enhancing the properties of photo-generated metallized nanocomposite coatings through thermal annealing

Marine Dabert [a], Dorina T. Papanastasiou [b], Loïc Vidal [c], Samar Hajjar-Garreau [c], Daniel Bellet [b], Daniel Lougnot [d], Lavinia Balan [a]*

[a]Univ. d'Orléans, Conditions Extrêmes Matériaux Haute Température et Irradiation CNRS UPR 3079, F-45000, Orléans, France

[b]Univ. Grenoble Alpes, CNRS, Grenoble INP, LMGP, F-38000 Grenoble, France.

[c]Univ. de Haute Alsace, Institut de Science des Matériaux de Mulhouse CNRS UMR 7361, F-68100, Mulhouse, France

[d]Univ. de Haute Alsace, Centre de Recherches sur les Economies, les Sociétés, les Arts et les Techniques CRESAT EA 3436, F-68100 Mulhouse, France.

*Corresponding author: E-mail: lavinia.balan@cnrs-orleans.fr; Tel: +33(0)238255655

1. Surface plasmon band observed for the 10 μm thick Ag@polymer coatings

UV-Vis spectroscopy shows that the as-synthesized 10 μm sample exhibits a surface plasmon band with a characteristic maximum at 460 nm. This phenomenon is directly related to the presence of silver nanoparticles and their specific morphology.

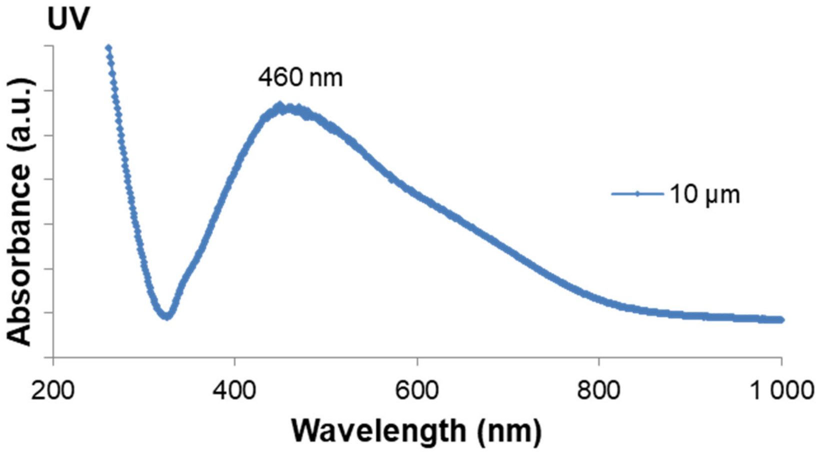

Figure S1. UV-vis spectrum of a 10 μm sample after UV synthesis.

2. X-ray diffraction (XRD) of Ag@polymer coatings

The intensity of each of the diffraction peaks associated with the Miller indices h k l is noted as $I_{h\,k\,l}$. This intensity can be compared to that of a randomly oriented sample (i.e. a powder). For the latter, the International Centre for Diffraction Data (ICDD) file 00-004-0783 associated with pure silver can be considered, its intensity being noted as $I_{0,h\,k\,l}$. The associated texture coefficients $C_{h\,k\,l}$ can be calculated for each family of crystallographic planes h k l as follows:

$$C_{h\,k\,l} = \frac{\frac{I_{h\,k\,l}}{I_{0,h\,k\,l}}}{\frac{1}{N}\cdot\sum\frac{I_{h\,k\,l}}{I_{0,h\,k\,l}}} \quad (1)$$

where N is the number of peaks considered (here N=4). For a randomly oriented sample, the texture coefficient of each peak is equal to unity. On the other hand, for a perfectly oriented

sample, the texture coefficients are equal to zero, except for the preferential orientation where Ch k l is equal to N. The texture coefficients clearly define which crystallographic planes are mainly observed. In addition, the texture coefficients can also be used to calculate the degree of preferential orientation σ of the specimen, defined as follows:

$$\sigma = \frac{\sqrt{\Sigma(C_{h\,k\,l} - 1)^2}}{\sqrt{N}.} \quad (1)$$

where the summation is performed over all values of *h k l* triplets. The degree of preferential orientation σ is 0 for randomly oriented and $\sqrt{N-1}$ for perfectly oriented ones.

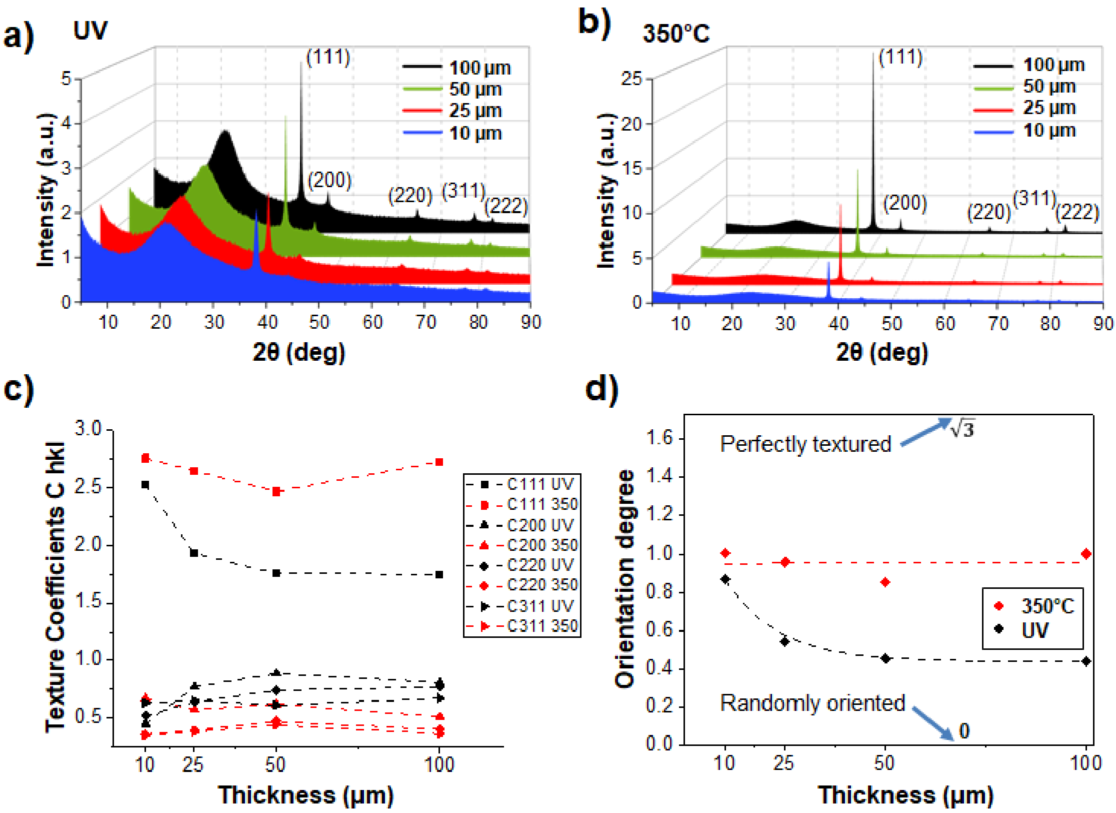

Figure S2. X-ray diffraction (XRD) analysis: a) X-ray diffraction patterns of Ag@polymer samples after synthesis (UV). b) after annealing (350 °C). c) Texture coefficients of the four main peaks (C111, C200, C220 and C311) vs. coating thickness. d) Degree of orientation vs. coating thickness after UV synthesis and annealing.

3. X-Ray Photoelectron Spectroscopy (XPS) of the 10 µm thick Ag@polymer coating

Important changes at the surface of the samples were revealed by XPS analysis. First of all, the amount of silver on the surface increased significantly after thermal annealing and the silver oxide disappeared, reduced by heating. Moreover a post polymerization occurred during annealing with the conversion of C=C double bonds to C-C single bonds.

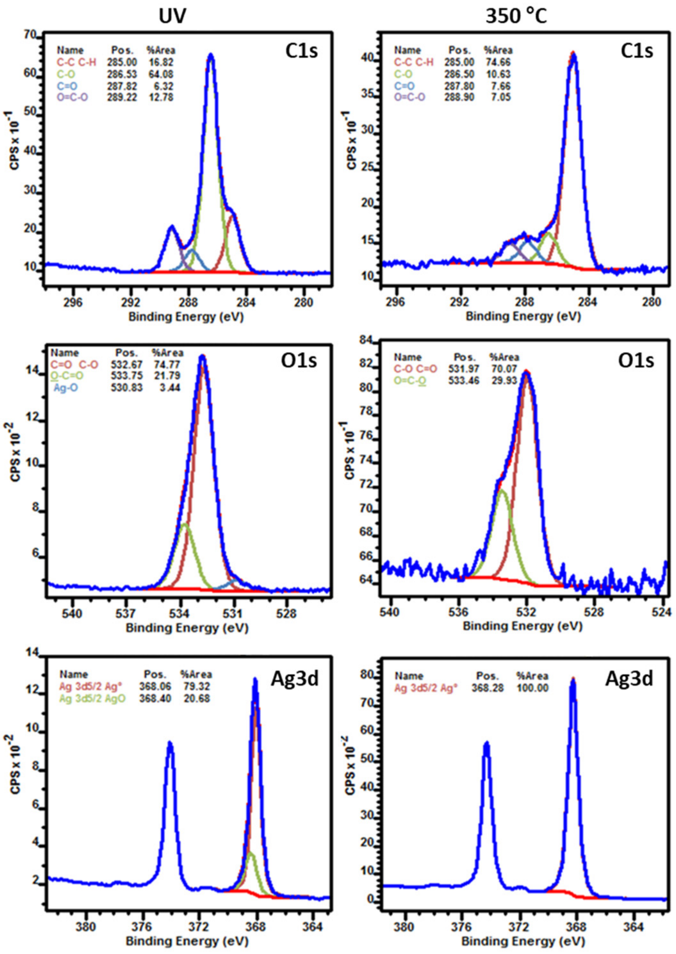

Figure S3. XPS analysis after UV synthesis (left column, UV) and after annealing treatment (right column, 350 °C) of a 10 µm sample – focus on the main elements (C, O and Ag).
